# Iron status is inversely associated with dietary iron intakes in patients with inactive or mildly active inflammatory bowel disease

**DOI:** 10.1186/1743-7075-10-18

**Published:** 2013-02-01

**Authors:** Jonathan J Powell, William B Cook, Mark Chatfield, Carol Hutchinson, Dora IA Pereira, Miranda CE Lomer

**Affiliations:** 1 MRC Human Nutrition Research, Elsie Widdowson Laboratory, Fulbourn Road, Cambridge, CB1 9NL, UK; 2 Department of Gastroenterology, Guy’s and St Thomas’ NHS Foundation Trust, London, UK; 3 King’s College London, Nutritional Sciences Division, London, UK

**Keywords:** IBD, Iron deficiency anaemia, Iron intake, Diet, Food frequency questionnaire

## Abstract

**Background:**

Patients with inflammatory bowel disease (IBD) frequently appear iron deplete but whether this is a reflection of dietary iron intakes is not known.

**Methods:**

Dietary data were collected from 29 patients with inactive or mildly-active IBD and 28 healthy controls using a validated food frequency questionnaire that measured intakes of iron and its absorption modifiers. Non-haem iron availability was estimated using a recently developed algorithm. Subjects were classified for iron status based upon data from a concomitant and separately published study of iron absorption. Absorption was used to define iron status because haematological parameters are flawed in assessing iron status in inflammatory conditions such as IBD.

**Results:**

Dietary intakes of total iron, non-haem iron and vitamin C were significantly greater in IBD patients who were iron replete compared to those who were iron deplete (by 48%, 48% and 94% respectively; *p*≤0.05). The predicted percentage of available non-haem iron did not differ between these groups (19.7 ± 2.0% *vs* 19.3 ± 2.0% respectively; *p*=0.25). However, because of the difference in iron intake, the overall amount of absorbed iron did (2.4 ± 0.8 mg/d *vs* 1.7 ± 0.5 mg/d; *p*=0.013). No such differences were observed in the healthy control subjects.

**Conclusions:**

In IBD, iron status is more closely related to the quality and quantity of dietary iron intake than in the general healthy population.

## Background

Iron deficiency is common in patients with inflammatory bowel disease (IBD) (36- 90% prevalence [1]), a diagnosis that encompasses Crohn’s disease and ulcerative colitis, and contributes to a reduced quality of life [2]. In IBD, protein and blood loss in the gastrointestinal tract contribute to abnormal iron loss, especially during inflammatory episodes, but dietary intakes of iron are also low, at least in subjects with Crohn’s disease [3]. This latter finding probably results from post-disease dietary changes, notably a reduction in the intake of fibrous foods, such as certain breakfast cereals that may be fortified with iron [3]. It is plausible that reduced intakes of both fibrous foods [3] and fortificant iron [Powell et al. 2012 submitted] provide some relief in gastrointestinal symptoms in IBD patients. Nonetheless, whether low dietary iron intakes are associated with iron deficiency in subjects with IBD has not been assessed. This, however, is potentially important. Patients with IBD may be especially sensitive to the gastrointestinal, adverse effects of oral iron supplements such that intravenous iron supplementation has been advised in clinical guidelines as a ‘preferred route’ in this population [2]. Thus intravenous iron replacement and/or adverse effects could be spared if appropriate dietary guidelines can be developed for IBD patients that (a) help to reduce the risk of iron deficiency developing and (b) do not themselves contribute to adverse effects through enhanced intake of redox-active fortificant iron [Powell et al. 2012 submitted].

However, addressing the question of whether dietary habits are associated with iron deficiency anaemia in this population has been complicated by two issues: first, in the absence of frank anaemia, routine measures of iron status are flawed in IBD- presumably because even low grade inflammation can influence haematological indices [4,5]. Secondly, iron intakes are a poor marker of iron exposure (i.e. absorption), chiefly because certain other dietary factors can markedly inhibit or promote iron absorption by altering its availability (i.e. the fraction of ingested iron that is at least potentially available for absorption from the intestinal lumen). In this study we have addressed both of these issues. First, as part of a broader programme investigating iron intakes, absorption, and quality of life in patients with IBD, iron deplete and iron replete subjects could be genuinely separated, based upon absorption of iron from a single oral dose rather than conventional haematological parameters [4]. Secondly, using an iron-specific, validated food frequency questionnaire and a recently developed algorithm, we were able to calculate ‘available iron’ hence taking account of absorption modifiers of dietary iron as well as total dietary iron [6,7]. We also studied a control group of healthy individuals, thus determining whether our findings would be IBD-specific or reflected in the general population as well as providing a comparison group for the data.

## Subjects and methods

### Study design and participants

IBD patients (*n*=29; 5 with ulcerative colitis and 24 with Crohn’s disease) were recruited from gastrointestinal outpatient clinics at Guy’s and St Thomas’ NHS Hospital Trust (GSTT), London, UK. Control subjects (*n*=28) were recruited from a local newspaper advertisement. Guy’s and St Thomas’ Local Research Ethics Committee approved the study. The data presented in this paper were collected as part of a larger programme and further details including the absorption data have been previously published [4].

### Patients

Patients, aged 18 to 65, were recruited from outpatient gastroenterology clinics at GSTT and in all cases IBD was diagnosed by histological and/or radiological techniques. Patients with other chronic diseases, pregnant and lactating females and those who had received iron therapy within the previous 28 days were excluded. Additionally, only patients with inactive or mildly active disease (e.g. Harvey Bradshaw Index < 8) were recruited for the study. Patients fulfilling these criteria were invited to participate in the study. All recruited subjects had a blood sample taken to assess traditional markers of iron status (full blood count, ferritin, serum iron, and total serum iron binding capacity) and inflammatory status (erythrocyte sedimentation rate, C-reactive protein), and these data are presented elsewhere [4].

### Controls

Potential subjects were recruited from an advertisement placed in a freely available newspaper distributed predominantly within Greater London. Subjects were initially screened by telephone to exclude anyone with known chronic disease, gastrointestinal disease, hereditary disorders of iron metabolism and those taking proton-pump inhibitor medication or iron therapy/supplements within the previous 28 days, as all these can act as confounders. Pregnant and lactating women were also excluded.

### Dietary intake: data collection and data output

A specific iron food frequency questionnaire (FFQ) was used to assess intakes of dietary iron and iron absorption modifiers over the previous month. The FFQ was a validated computerised quantitative food frequency questionnaire that had been developed specifically for iron and validated against weighed diet records collected over 11 days [6]. Usually the FFQ is self-completed by the volunteer on a computer. However, due to restricted access for non-hospital staff to computing facilities, it was necessary to modify the administration of the questionnaire. Rather than the subject directly entering their dietary intake into a computer program the data were collected and recorded manually by one of the authors using the same prompts and questions as the computer program.

Completion of this iron specific food questionnaire entailed 4 steps : (i) overall meal frequency recall (ii) individual meal frequency reporting (iii) ‘missing foods’ checklist and (iv) confirmation of the information provided. Specifically, we :

(i) recorded how many times per week subjects ate any meal or snack (e.g. breakfast 7 times per week).

(ii) used a list of 630 foods, divided into sixteen groups, to ascertain the meals and snacks that subjects had consumed in the previous month. In addition, subjects reported the portion sizes and the frequency of each meal or snack per week. To aid estimation of portion sizes, food models and a photographic food portion atlas [8] were used. Subjects were asked to list the ingredients and quantities used in composite dishes.

(iii) presented to the subjects a checklist of eighty foods pertinent to iron intake or iron absorption that was completed to capture any foods missing from the diet recall so far.

(iv) presented the subjects with their diet report and asked them to confirm its accuracy or correct it.

Following the collection of dietary data from the subject, the information was entered into the computerised iron FFQ program. The foods within the database (i.e. the 630 in the FFQ) were only those commonly found in the Western diet that contain iron (or zinc ^a^), or a dietary component that modifies iron absorption such as vitamin C, animal tissue (i.e. red meat, fish or poultry), phytate, calcium, alcohol, tea and coffee. Thus not *all* foods eaten by subjects were captured by the FFQ or, therefore, entered into the FFQ program.

Average daily intakes were calculated using a Microsoft Excel based computer program (MBIAT version 4.2, Institute of Food Research, Norwich, UK) for the following dietary components: total iron, non-haem iron, haem iron, meat iron, vitamin C, phytate, calcium, meat/fish/poultry, black tea equivalents and alcohol. Values for total iron, vitamin C and calcium were calculated from the UK Food Composition Tables and Supplements [9-16]. Meat/fish/poultry values represent the amount of animal tissue in 100 g edible portion of food. The amount of haem iron was calculated from the product of the meat iron content and the proportion of haem iron in the specific meat from literature values [17,18]. Accordingly, non-haem iron values were calculated as the difference between haem iron and total iron. Phytate values were calculated from published data [13,15,18-21]. Finally, black tea equivalents were estimated from beverages containing a significant tannin (iron-binding polyphenol) content based on their inhibitory effect on iron absorption from published data [22-24]. 100 g of black tea was assigned an arbitrary value of 100 and other beverages containing tannins were assigned a proportion of this value dependent upon their inhibitory effect on iron absorption in comparison to black tea.

### Dietary intakes: estimation of available (non-haem) iron

Estimated availability of dietary non-haem iron was calculated using the recently developed algorithm of Rickard *et al. *[7] namely:

$$\begin{array}{l} \%\;\text{Available}\;\text{Non}-\text{Haem}\;\text{Iron}\\ =22.42*\frac{\left(1+\ln\left(1+0.0056*\mathrm{AA}\right)\right)*\left(1+\ln\left(1+0.0008*\mathrm{AT}\right)\right)}{\left(1+\ln\left(1+0.0008*C\right)\right)*\left(1+\ln\left(1+0.0033*P\right)\right)*\left(1+\ln\left(1+0.0004*\mathrm{PO}\right)\right)*\left(1+\ln\left(1+0.0424*\mathrm{NH}\right)\right)} \end{array}$$

where: AA, ascorbic acid (mg); AT, red meat, fish and poultry (g); C, calcium (mg); P, phytate (mg); PO, polyphenols from tea (mg); NH, dietary non-haem iron (mg).

The availability of dietary non-haem iron was calculated for six meal events per subject (breakfast, morning snack, lunch, afternoon snack, evening meal and evening snack) based on their average monthly dietary intake at each of these meals and then an average was taken from these to give mean daily non-haem iron availability. We did not consider the ‘available total dietary iron’ (i.e. available non-haem + available haem iron) because ‘available haem iron’ is typically ascribed a standard percentage (e.g. 25%) of total haem iron intake irrespective of other dietary variables [7]. Thus, in comparison between groups, total haem iron entirely reflects ‘available haem iron’, unlike the situation described herein for non-haem iron.

### Classification of iron deplete and iron replete

Subjects were classified as iron deplete or replete based upon the serum iron curves obtained following ingestion of a single 200 mg ferrous sulphate capsule (65 mg Fe) [4]. Based upon findings in healthy volunteers [4], iron absorbers were defined as having a rise in serum iron greater than 5 μmol/L from baseline in a four-hour period post-iron ingestion whilst iron non-absorbers had a serum iron rise of less than 5 μmol/L. This classification was used to identify iron replete and iron deplete subjects as traditional clinical haematological parameters (ferritin, transferrin saturation and even soluble transferrin receptor) are not reliable indicators of iron status in IBD patients due to being acute phase reactants [4,5] and, apart from bone marrow measurements, iron absorption is the best measure of iron status [25,26].

### Statistical analysis

The primary objective was to compare dietary intakes of iron and available non-haem iron between IBD patients that were iron deplete and iron replete. Due to the limited numbers that can practically be assessed for iron status we have presented statistically unadjusted data (i.e. not adjusted for multiple comparisons). We present the same data for control subjects as a reference group and have also presented a comparison of dietary intakes between IBD patients and controls allowing comparison to previous work [3]. Statistical comparisons were by the Mann–Whitney test and significance was assumed at *p* ≤ 0.05. Values expressed are median, interquartile range (IQR) and range unless stated otherwise.

## Results

A total of fifty-seven subjects (IBD n=29, controls n=28) were included in the study. Controls (twelve males) had a mean age of 35 (SD=11) years and BMI of 23.4 (SD=3) kg/m^2 ^and IBD patients (thirteen males) had a mean age of 42 (SD=13) years and BMI of 25.7 (SD=6) kg/m^2 ^[4].

### Dietary intakes

Table 1 shows the dietary intakes for IBD patients and healthy controls. The phytate intake was significantly lower in the patient group (*p*<0.001), whereas the tea consumption was significantly higher (*p*=0.05) for this group when compared to the healthy control group. No other significant differences were observed in the nutrients investigated with our FFQ.

**Table 1:** **Dietary intakes in IBD patients and controls measured using an iron-specific FFQ**[6]

	**Patients (*n *=29)**			**Controls (*n *=28)**			***p-value***
	**Median**	**IQR**	**Range**	**Median**	**IQR**	**Range**	
**Total dietary iron (mg/d)**	11.5	8.7-14.3	4.7-27.0	10.7	8.3-14.5	4.5-44.3	0.80
**Non-haem iron (mg/d)**	11.2	8.1-13.7	4.3-25.1	10.5	7.8-13.8	4.2 -42	0.80
**Haem iron (mg/d)**	0.5	0.4-0.9	0-1.85	0.5	0.2-0.7	0-2.4	0.5
**Meat, fish and poultry (g/d)**	129	94-166	0-360.4	109	57-144	0-435	0.2
**Vitamin C (mg/d)**	74	51-113	7.4-192.7	83	61-161	13.4-315.2	0.14
**Phytate (mg/d)**	46	32-63	7.1-209.9	97	67-119	23-439.7	<0.001
**Black tea equivalents (g/d)**	583	432-1005	0-1909.71	412	118-732	0-1718.6	0.05
**Calcium (mg/d)**	905	650-1112	223.6-1914.4	645	475-971	158.7-2251.1	0.06
**Alcohol – n (%) consumers**	15 (52%)			20 (71%)			
**Alcohol – (g/d) consumers**	7	2-13	0-90.7	10	4-17	0-49	0.08
^**(1)**^**Predicted available non-haem iron (mg/d)**	1.9	1.5-2.4	0.8-4.1	1.9	1.5-2.5	0.9-4.1	0.8

Abbreviations: *IQR* interquartile range; *IBD* inflammatory bowel disease; *FFQ* food frequency questionnaire; (1) Predicted from Rickard *et al.*[7].

However, when we classified the IBD patients as iron deplete or iron replete we observed significant differences in mean daily intakes for total dietary iron (*p*=0.05), non-haem iron (*p*=0.045), and vitamin C (*p=*0.017), with the iron replete group having a greater intake of these nutrients (Table 2). In contrast, the healthy control iron replete subjects had a higher overall intake of meat and fish products (*p*=0.005) that was reflected in a higher intake of haem iron (*p*=0.03), compared to the iron deplete healthy subjects (Table 2).

**Table 2:** Dietary intakes in patients and controls classified as iron deplete or iron replete^*^

	**IBD Patients (*n*=29)**		**Controls (*n*=28)**			
	**Iron deplete (*n*=21)**	**Iron replete (*n*=8)**	**Iron deplete (*n*=18)**	**Iron replete (*n*=10)**	**IBD Patients**	**Controls**
	**Median**	**Median**	**Median**	**Median**	***p- *value †**	***p- *value †**
**Total dietary iron (mg/d)**	10.0	14.8	11.2	10.3	0.05	0.7
**Non-haem iron (mg/d)**	9.4	13.9	10.8	9.8	0.045	0.8
**Haem iron (mg/d)**	0.5	0.8	0.4	0.7	0.13	0.03
**MFP (g/d)**	120	177	82	143	0.07	0.005
**Vitamin C (mg/d)**	66	128	83	104	0.017	0.7
**Phytate (mg/d)**	48	44	93	111	0.7	0.3
**BTE (g/d)**	731	472	392	440	0.3	0.9
**Calcium (mg/d)**	927	899	645	615	0.6	0.8
**Alcohol - n (%) consumers**	10 (48%)	5 (63%)	11 (61%)	9 (90%)		
**Alcohol - (g/d) consumers**	7.8	5.6	10.3	8.9	0.7	0.13
^**(1)**^**predicted available non-haem iron (mg/d)**	1.7	2.4	1.9	1.8	0.013	0.5

Abbreviations: *MFP* meat,fish and poultry; *BTE* black tea equivalents; *IBD* inflammatory bowel disease; (1) Predicted from Rickard *et al.*[7].

* ‘Iron deplete’ refers to subjects who significantly absorbed iron when tested while ‘iron replete’ refers to those who did not (see Methods section).

† Compares dietary intakes in iron deplete and iron replete for IBD patients or controls.

### Estimation of available non-haem

**Figure 1:**
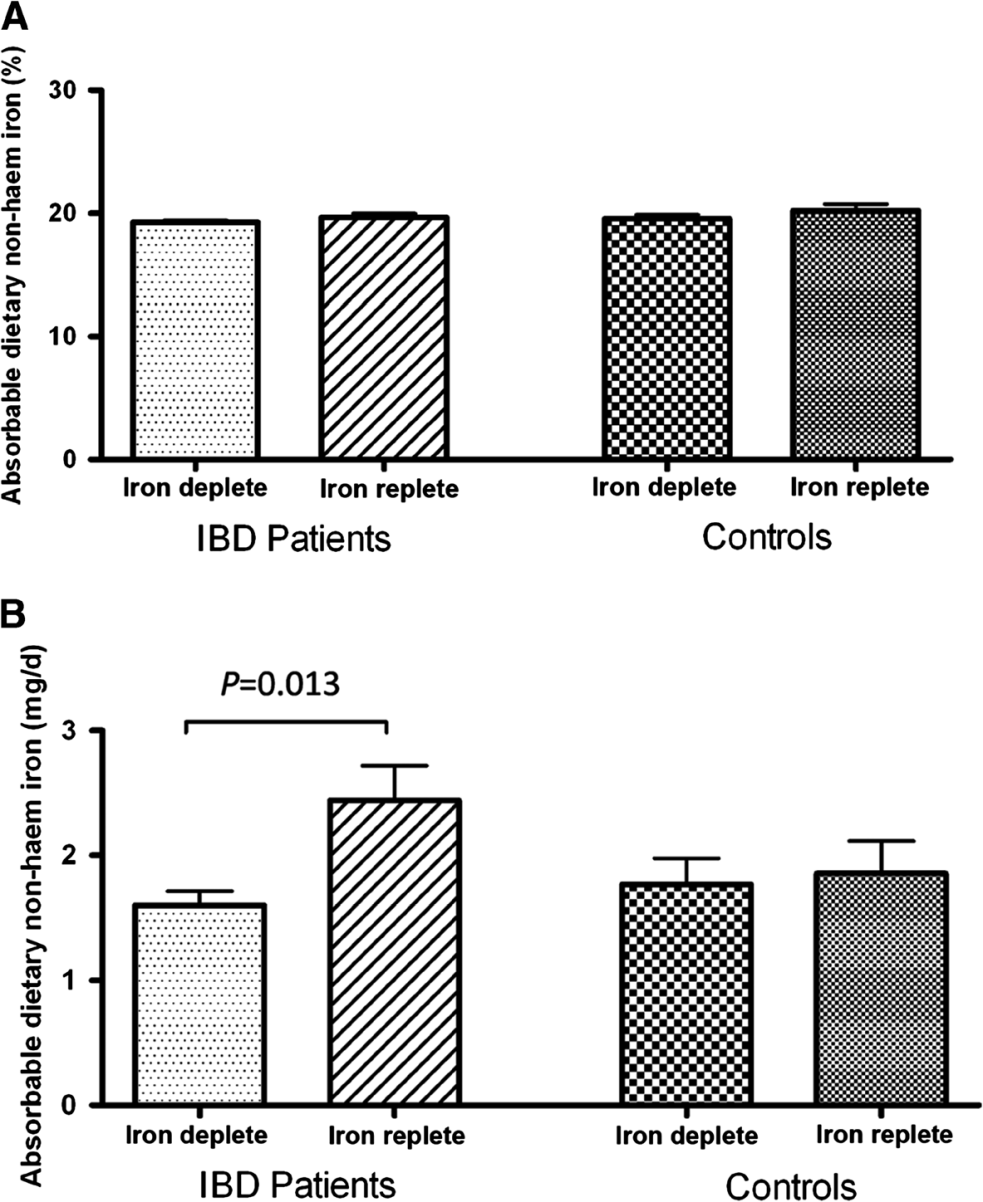
**Absorbable dietary non-haem iron.** Absorbable non-haem iron was calculated using the algorithm of Rickard *et al.*[7]. Results are shown as mean ± SEM (**A**) expressed as a percentage and (**B**) as absolute values calculated from non-haem iron intakes (shown in Table 2). IBD, inflammatory bowel disease.

The predicted *percentage* of available non-haem iron, calculated using the Rickard *et al.* algorithm [7] did not differ between iron deplete and iron replete subjects both in the patient and control groups (Figure 1A). On the contrary, the *absolute quantity* of non-haem iron available for absorption was significantly lower (*p*=0.013) in iron deplete IBD patients compared to iron replete IBD patients (Figure 1B), chiefly due to the difference in non-haem iron intake between the 2 groups. No significant difference in the absolute quantity of non-haem iron available for absorption was observed in the healthy control subjects (*p*=0.5) (Figure 1B).

## Discussion

We sought to determine here whether quality and/or quantity of dietary iron ingested was associated with iron status in patients with IBD, even if in the general population such associations are difficult to detect [27,28]. The idea that dietary iron intakes may be more of a determinant of iron status in IBD subjects than the normal healthy population was based on several factors. In particular, IBD patients are frequently iron deplete placing heavy emphasis upon adequate iron intakes and availability. In addition, disease symptoms may lead to dietary recommendations/ modifications thus altering intakes of iron or nutrients that can modify iron absorption [3,4]. The findings presented here clearly illustrate that iron deplete IBD patients have a history of low non-haem iron intakes but no difference in percentage availability compared to iron replete subjects. This is in contrast to healthy subjects who, in this study and, as noted above, elsewhere [27,28], seem to be less sensitive to modest changes in non-haem iron intakes/availability in relation to iron status. Nonetheless, it should be noted that haem iron intakes *did* differ for iron replete versus iron deplete control subjects, so it is possible that this, rather than non-haem iron, contributed to differences in iron status.

**Figure 2:**
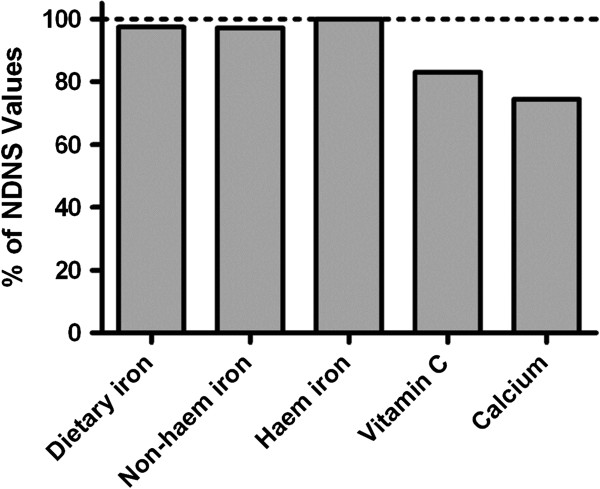
**Dietary intakes as a percentage of national averages.** Mean dietary data of control subjects shown as a percentage of the mean values for intakes of males and females from the U.K. National Diet and Nutrition Survey (NDNS).

The finding that non-haem dietary iron intakes may help determine iron status in patients with IBD could stimulate further thinking on how IBD patients are best advised to maximise dietary iron intakes. However, care must be exercised in this process. Firstly, as noted above, data from this same cohort also imply that high fortificant iron intakes actually impact quality of life in a *negative* fashion [Powell et al. 2012 submitted], perhaps due to direct, adverse gastrointestinal effects of certain forms of iron that are not naturally derived from the diet [29-31] [Powell et al. 2012 submitted]. Secondly, although the iron FFQ used in this study is a validated method for collecting dietary data with respect to iron and its absorption modifiers [6], like all forms of dietary assessment it is not immune to measurement error. The iron FFQ does not provide data on energy intake that could then be used to identify likely under reporters. However, comparison of the dietary data collected from control subjects in this study with data from The UK National Diet and Nutrition Survey, 2003, shows good agreement for intakes of total dietary iron, non-haem iron and haem iron, and reasonable agreement for vitamin C and calcium (Figure 2). Thirdly, numbers in this study were relatively small, being constrained by the necessity for individual iron absorption tests. Fourthly, we did not observe lower iron intakes in patients with IBD compared to controls, despite a much larger study reporting this to be the case [3]. In fairness, our aims were not to replicate these previous findings, and the study was not powered, or indeed matched (e.g. by age), to do so, but, rather, we wished to assess how iron intakes were associated with iron status in IBD. Nonetheless, it is worth noting that this study does echo some similar findings to those of the larger study [3], in that IBD patients had lower intakes of vitamin C and phytate in comparison to healthy controls.

Thus, in conclusion, overall our data indicate that iron status is more closely related to dietary intake in patients with IBD than the general healthy population. However, care must be taken in advice given to patients on modifying their diet to enhance dietary iron intakes as our studies on quality of life, concurrent with this work, suggest that elevated fortificant iron intakes may be associated with a reduced quality of life in patients with IBD. The risk:benefit ratio of low versus high dietary iron deserves further investigation in this patient group.

## Endnote

^a^Note: Zinc intake is also captured by this FFQ but was not a focus of our studies.

## Abbreviations

IBD: Inflammatory bowel disease; GSTT: Guy’s and St Thomas’ NHS Foundation Trust; IQR: Interquartile range; FFQ: Food frequency questionnaire.

## Competing interests

The authors have no competing interest.

## Authors’ contributions

The authors’ responsibilities were as follows: MCEL and JJP designed the study. MCEL and WBC carried out the study. JJP, MC, WBC, DIAP and CH carried out data analysis. All authors have contributed to the preparation of the manuscript and have approved the manuscript. JJP and DIAP had primary responsibility for the final content of the manuscript.

## Authors’ information

Jonathan J Powell and William B Cook equal first authorship.
